# Side-Polished Fiber-Optic Line Sensor for High-Frequency Broadband Ultrasound Detection

**DOI:** 10.3390/s19020398

**Published:** 2019-01-18

**Authors:** Jeongmin Heo, Kyu-Tae Lee, Ryun Kyung Kim, Hyoung Won Baac

**Affiliations:** 1 Department of Electrical and Computer Engineering, Sungkyunkwan University, Suwon 16419, Korea; gofal114@skku.edu (J.H.); rkkim@skku.edu (R.K.K.); 2 Department of Physics, Inha University, Incheon 22212, Korea; ktlee@inha.ac.kr

**Keywords:** ultrasound sensor, fiber-optic sensor, optical ultrasound detector, side-polished fiber, high-frequency ultrasound

## Abstract

We demonstrate a side-polished fiber-optic ultrasound sensor (SPFS) with a broad frequency bandwidth (dc–46 MHz at 6-dB reduction) and a wide amplitude detection range from several kPa to 4.8 MPa. It also exhibits a high acoustic sensitivity of 426 mV/MPa with a signal-to-noise ratio of 35 dB and a noise-equivalent pressure of 6.6 kPa (over 1–50 MHz bandwidth) measured at 7-MHz frequency. The SPFS does not require multi-layer-coated structures that are used in other high-sensitivity optical detectors. Without any coating, this uses a microscale-roughened structure for evanescent-field interaction with an external medium acoustically modulated. Such unique structure allows significantly high sensitivity despite having a small detection area of only 0.016 mm^2^ as a narrow line sensor with a width of 8 μm. The SPFS performance is characterized in terms of acoustic frequency, amplitude responses, and sensitivities that are compared with those of a 1-mm diameter piezoelectric hydrophone used as a reference.

## 1. Introduction

Optical ultrasound sensors have been actively studied to achieve high sensitivity and broad acoustic frequency bandwidth. It has been demonstrated that a variety of interferometric sensors have such characteristics. For example, polymer microring resonators [1,2,3,4], Fabry-Perot etalons [5,6,7], and Mach-Zehnder structures [8,9]. Among them, the recent optical ring resonators exhibited a low noise equivalent pressure of 105 Pa over 350-MHz bandwidth [2]. The Fabry-Perot sensors have particular advantages in terms of fabrication flexibility. Their multi-layer structures can be produced on a glass substrate [5,10,11], as well as on a tiny microscale tip of optical fibers to realize miniature ultrasound devices [12]. Furthermore, these structures have been utilized for all-optical transducers with not just a single element [12,13] but also with an optically addressable multi-channel configuration [14]. Despite these advantages, such polymer microrings and multilayer-coated Fabry-Perot interferometers are not mechanically robust when exposed to high-amplitude acoustic pressure of more than a few MPa. Moreover, the fragile coatings are not suitable for detecting acoustic cavitation that can cause physical damage.

For optical measurements of high-amplitude acoustic pressure, fiber-optic sensors with a flat cleaved edge have been widely utilized without any coatings have been used instead [15,16,17,18,19]. These rely on a single optical reflection at a sensor interface in contact with water. These robust sensors allowed to characterize focused ultrasound amplitudes for tissue therapy up to tens of MPa [18,19,20,21] with a sensitivity of 1–10 mV/MPa [22].

A side-polished fiber (SPF) has provided a useful platform for a variety of optical sensors based on surface plasmon resonance (SPR) [23,24,25], Bragg grating [26,27] and polymer waveguide [28]. A mechanical polishing technique was used to partially remove a glass cladding region, only leaving an extremely thin cladding layer (<1 μm) above the fiber core. For example, a silver-coated SPF has been developed as an SPR-based biosensor with a high sensitivity of 4365.5 nm/refractive index unit (RIU) providing an optimal detection range of 1.38–1.40 RIU [24]. 

We demonstrate a side-polished fiber sensor (SPFS) for ultrasound detection with a frequency bandwidth up to 46 MHz, an amplitude dynamic range of several kPa–4.8 MPa, an acoustic sensitivity of 426 mV/MPa at 7 MHz, and a noise-equivalent pressure (NEP) of 6.6 kPa (over 1–50 MHz bandwidth) obtained at 7 MHz. Our SPFS has a particular optical architecture with the upper cladding region above the core entirely removed. Its partially polished core with microscale-roughened surface is exposed directly to an external medium. There is no additional coating on the polished region, making the structure mechanically robust and reliable against high-pressure amplitudes of several MPa. The SPF works as a narrow line form (8 μm) of an acoustic sensor based on evanescent-field interaction along the polished fiber region, enabling significantly higher sensitivity than other non-coated optical sensors. 

## 2. Materials and Methods 

**Figure 1:**
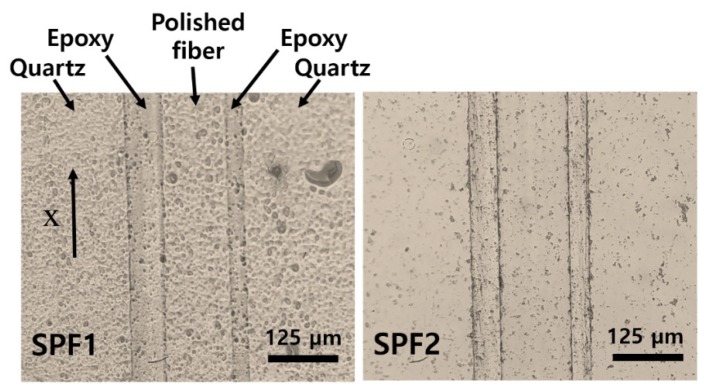
Top views of two side-polished fiber (SPF) sensors with different surface roughness. The left arrow indicates the direction of x-axis defined here as parallel to the longitudinal direction of fiber.

A single-mode optical fiber (SMF28; Corning, USA) was mounted on a longitudinal groove (V-shaped) formed in a quartz block and then fixed using a UV-curable epoxy. Here, the groove was slightly bent with 250-mm radius of curvature, resulting in a small bent angle of α (= 0.114°; configuration described in Section 3). The bent polished fiber could lead to an effective optical interaction region of a few mm in length [29]. The fiber was polished with alumina powders (particle size: 4 μm) for a hard grinding process and then with cerium oxide powders (particle size: 2.5 μm) for softening the surface [29]. These steps removed the top cladding entirely and the core partially, producing microscale roughness on the polished core. We prepared two sensors (SPF1 and SPF2) with different roughness as shown in Figure 1a,b. The core thickness after polish was calculated as 4 and 3.8 μm, respectively. The same hard grinding condition in terms of strength and process time was used for both sensors, but SPF2 was smoothened further by increasing the softening step period. In SPF1, the lateral dimension of rough particles was in the order of tens of μm, while SPF2 had a relatively smooth surface with several-μm particles. We confirmed high optical losses through scattering at the roughened interface. When the sensors were contacted with water, input and output power ratios through SPF1 and SPF2 (10 log P_in_/P_out_) were 31 and 22.6 dB, respectively.

**Figure 2:**
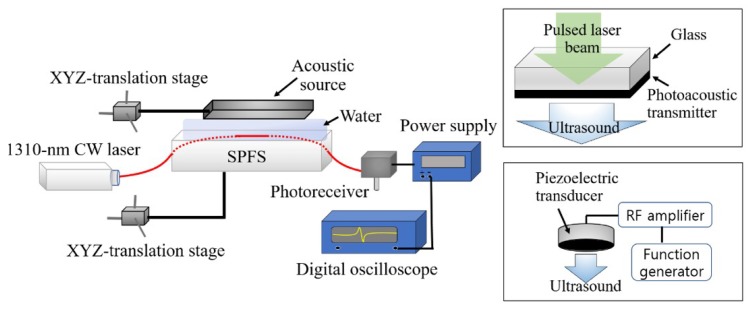
An experimental setup for sensor characterization. Two types of acoustic sources were used to characterize the side-polished fiber sensor (SPFS). The top inset box shows a schematic to use a photoacoustic transmitter (Cr or CNT-PDMS film) as the source, and the bottom inset box to use a piezoelectric transducer.

The SPFS was operated by using a continuous-wave (CW) probe laser beam (1310-nm wavelength; Thorlabs, USA) as an input, as shown in Figure 2. The output optical signal was detected by a broadband photoreceiver (125-MHz bandwidth; Newport, USA). Two types of acoustic sources were used to characterize the SPFS, shown by two inset boxes in Figure 2. As an acoustic input for broadband characterization, we used thin-film photoacoustic transmitters. Both carbon nanotube (CNT)-polydimethylsiloxane (PDMS) composite with > 30 μm thickness and Cr coating having 100 nm thickness, shown by the top inset box in Figure 2 [30,31]. For a plane wave configuration, these transmitters were closely positioned with 1.5-mm distance from the SPFS. Under pulsed laser irradiation (532-nm wavelength and 6-ns width; Litron laser, UK), these transmitters could produce short ultrasonic pulses replicating the temporal shape of an incident optical pulse [30,32]. For comparison, we used a calibrated polyvinylidene difluoride (PVDF) needle hydrophone with 1-mm diameter (Precision Acoustics, UK) replacing the SPFS in Figure 2. 

Here, the CNT-PDMS transmitter was designed and fabricated to have a frequency bandwidth of <30 MHz, which is relatively lower than the one reported elsewhere [33]. However, such a bandwidth is still wide enough for characterization of the SPFS and the reference hydrophone.

## 3. Results and Discussion

**Figure 3:**
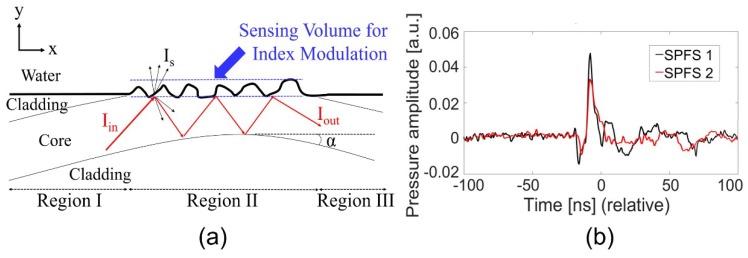
(**a**) A schematic diagram showing SPFS operation (α = 0.114°). The sensing volume increases with polish-induced roughness at the core surface. (**b**) Acoustic signal waveforms for two sensors with different roughness. The signal-to-noise ratio (SNR) increases with the roughness (in this example, 17.4 and 15.74 dB for SPF1 and SPF2, respectively).

The optical fiber can be divided into three parts along the longitudinal direction as demonstrated in Figure 3a. Region I represents a non-polished input part, region II a polished sensing region with the exposed fiber core, and region III a non-polished output part. A single-mode optical wave in region I is initially guided with a propagation angle of 85°. It then runs into the sensing surface at the entrance of region II, which is shown as I_in_. Here, the angle of incidence (85°) is greater than the critical angle at the core/water interface (θ_C_ = 67°). After passing through region II, it couples back to the non-polished region (region III) connected to the photoreceiver, which is shown as I_out_.

In the SPFS, the evanescent field of core mode interacts with the refractive index modulation of water in region II, although there are high optical losses via scattering (I_s_) and mode mismatch between regions. Such evanescent field interaction and thus the sensing process occurs over the entire volume of roughened structure, which is indicated by the thick arrow at the top of Figure 3a. More roughness plays a role of increasing effective depth and volume for index modulation. Therefore, we can enhance index sensitivity by increasing the volume of polish-induced roughness in region II. Figure 3b demonstrates that the signal-to-noise ratio (SNR) increases with the roughness. Here, we used a common source input (Cr film; photoacoustic transmitter excited by a laser energy of 10 mJ/pulse) to compare signal strengths from SPF1 and SPF2. In this example, SPF1 and SPF2 had the SNR values of 17.4 and 15.74 dB, respectively. The peak amplitude of SPF1 was 1.5 fold higher than that of SPF2. Note that the dc output powers of SPF1 and SPF2 were 16 and 105 μW for a common input power of 20 mW. This means that SPF1 can lead to stronger modulation of evanescent field than SPF2 when the refractive index of water is acoustically modulated, despite higher optical loss as confirmed by the dc output power. Due to the higher performance of SPF1 than SPF2, we used SPF1 only for the following characterization (named SPFS).

**Figure 4:**
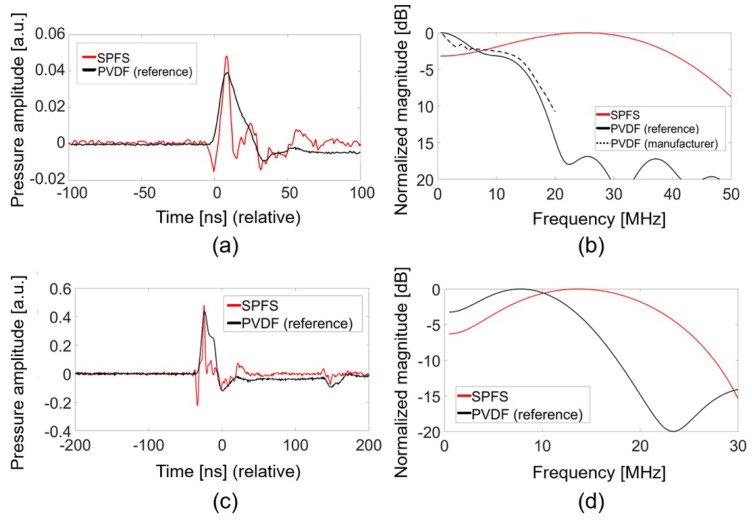
Acoustic signal waveforms and frequency spectra: (**a**,**b**) from the Cr film used as a source; (**c**,**d**) from the CNT-PDMS transmitter as a source.

We compared acoustic frequency characteristics of SPFS (=SPF1) and PVDF hydrophone under the same measurement conditions. For broadband frequency characterization, we first used the Cr transmitter capable of duplicating an incident Gaussian shape of laser pulse into an acoustic pulse. This generates narrow ultrasonic pulses (~7 ns) with a bandwidth from dc to 70 MHz (at 6-dB reduction) which are suitable to find a detector output close to an impulse response [30]. Figure 4a shows the measured temporal waveforms for the Cr film excited by a pulse laser energy (E) of 2.2 mJ. We assume these waveforms as impulse responses of detectors. The peak pressure amplitude of SPFS was 14% higher than that of the PVDF hydrophone. The SNR values of two waveforms were 13.5 and 14 dB for SPFS and PVDF hydrophone measurement, respectively. Here, two temporal waveforms were changed from the input Gaussian pulse shape, implying that the frequency bandwidths of two detectors are different. The frequency spectrum for the SPFS in Figure 4b demonstrates a peak response at 25 MHz and a wide frequency bandwidth over 46 MHz at the upper 6-dB roll-off point. We also obtained the characteristic spectrum of PVDF hydrophone (black solid line in Figure 4b) by using the Cr film as an approximate impulse response generator, which demonstrates the narrow bandwidth of only 16 MHz with a decreasing response from dc. This result almost agreed with the manufacturer-calibrated spectrum available up to 20 MHz only (black dotted line; Precision Acoustics, UK) [34]. 

Next, we used the CNT-PDMS transmitter as a source to compare the detector responses. It was significant to use CNT-PDMS transmitters because they are high-frequency and high-amplitude sources widely utilized for a variety of all-optical ultrasound transducers for imaging and therapy [20,30,31]. Figure 4c,d show that the SPFS produced a slightly higher peak pressure (9%) and a wider bandwidth than the reference case. The SNR values were 22.8 and 23.4 dB for waveforms measured by SPFS and PVDF hydrophone, respectively. The measurement with CNT-PDMS again confirms that the frequency spectrum of SPFS is enriched by higher-frequency components than that of PVDF hydrophone: 14 MHz at the peak and 26 MHz at the 6-dB drop point.

**Figure 5:**
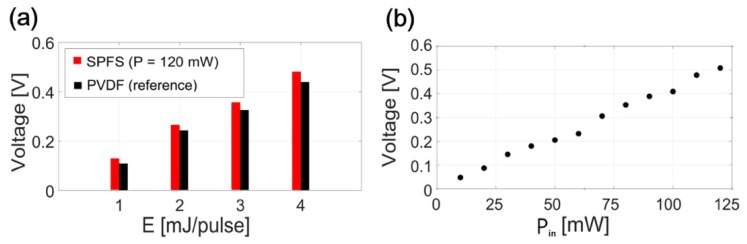
(**a**) Comparison of ultrasound signal amplitudes measured by SPFS and polyvinylidene difluoride (PVDF) hydrophone for the CNT-PDMS transmitter excited by E = 1, 2, 3 and 4 mJ/pulse); (**b**) SPFS peak signal values versus the probe laser beam power P_in_ (CNT-PDMS transmitter excited by E = 4 mJ/pulse).

From the CNT-PDMS, the measured peak pressure values were consistently higher for the SPFS as the input pressure increased as demonstrated in Figure 5a. The SPFS provided peak values of 0.13, 0.26, 0.36, and 0.48 V for E = 1, 2, 3 and 4 mJ/pulse, each of which was 15%, 12%, 12%, and 11% higher than the values measured by the reference detector (0.11, 0.23, 0.32, and 0.43 V), respectively. In the SPFS, the strength of an output signal increased with probe laser beam power (P_in_) as demonstrated in Figure 5b. For a fixed input pressure generated by the CNT-PDMS transmitter (E = 4 mJ/pulse), a linear increase trend was confirmed for P_in_ = 10–120 mW. The SPF output strength was limited by the maximum-available power of the probe laser source (120 mW).

**Figure 6:**
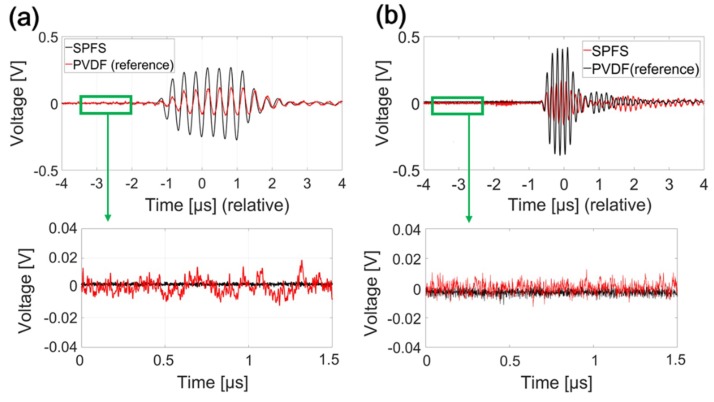
Temporal waveforms measured by SPFS and reference hydrophone for acoustic inputs with (**a**) 3-MHz and (**b**) 7-MHz frequencies (P_in_ = 120 mW). The bottom figures show enlarged views for early periods used for noise analysis (green boxes of the top figures) before the major signal traces arrive.

For sensitivity evaluation, we used two calibrated piezoelectric transducers with center frequencies of 3 and 7 MHz as acoustic sources, as demonstrated in Figure 6. Although 3- and 7-MHz frequencies are far from the central frequency response range of SPFS, we utilized them as acoustic inputs to provide frequency ranges commonly detectable by SPFS and PVDF hydrophone. Note that the SPFS responses at 3 and 7 MHz are 3- and 2.5-dB lower than that of the peak frequency (25 MHz). Here, the 3-MHz transducer has a planar shape (diameter: 15 mm) that was placed 1.5 mm apart from the detector surface. The 7-MHz source was slightly concave (diameter: 5 mm, radius of curvature: 12 mm) but was placed close to the detector (2 mm apart). Peak amplitudes measured by these calibrated references were 267.3 and 427.9 mV, corresponding to 270 and 400 kPa for 3 and 7 MHz sources, respectively.

In terms of peak signal amplitudes in Figure 6, the SPFS produced lower amplitudes of 112.2 and 173.6 mV, resulting in 417 and 426 mV/MPa at 3 and 7 MHz, respectively. The comparative study at 3 and 7 MHz frequency confirms that the peak amplitudes from the SPFS corresponded to 42.0% and 40.6% of those measured from the PVDF hydrophone. However, it should be noted that the SPFS has a small detection area of 0.016 mm^2^ calculated from 8.3-μm core thickness and 2-mm length. This corresponds to only 2% of the reference detector area (0.785 mm^2^). This confirms that the SPFS produces significantly higher output amplitudes despite the small sensing area. The acoustic sensitivity of 417–426 mV/MPa is significantly higher than those of other optical detectors designed for high-amplitude pressure measurement; for example, 6 mV/MPa for a commercially-available fiber-optic hydrophone [22] and 65.4 mV/MPa for an all-silica fiber-optic Fabry-Perot interferometer [35].

In order to determine noise levels of SPFS and PVDF hydrophone, we chose a 1.5-μs period shown in the bottom of Figure 6a,b, respectively, before the major signal traces arrive at the detectors. Peak signal amplitudes were determined from the top waveforms of Figure 6a,b. For the 3-MHz acoustic input, the SNRs of SPFS and PVDF hydrophone were 32 and 41 dB, respectively. For the 7-MHz case, they were 35 and 49 dB.

We also determined the NEP at 7 MHz. The rms noise voltage for the photoreceiver bandwidth (1–125 MHz) was first measured without any acoustic input. This value was then multiplied by a factor of 3 to determine the peak noise voltage that can be used as a realistic indication for broadband signal measurement [11]. By dividing the peak noise by the calibrated acoustic sensitivity (426 mV/MPa at 7-MHz frequency), we obtained 16.2 kPa over a 1–125 MHz bandwidth. For a 1–50 MHz bandwidth that is close to that of 6-dB bandwidth of SPFS, the NEP was reduced to 6.6 kPa. Similarly, we obtained the NEP values at 3-MHz frequency: 9.2 and 22.2 kPa for 1–50 and 1–125 MHz bandwidths. The NEP of SPFS is better than other results obtained over narrow bandwidths: 250 kPa for the single-mode fiber-optic detector (2-MHz bandwidth) [22], 19.4 kPa for the Fabry-Perot optical detector (2.5-MHz bandwidth) [35], and 15 kPa for the fiber-optic Fabry-Perot sensor (20-MHz bandwidth) [11].

For the above noise analysis using the 7-MHz concave source, we avoided choosing the crosstalk signal shown between −2 and −1 μs range in Figure 6b. The early-arriving crosstalk in the SPFS is caused by a geometrical shape of the 7-MHz source. Ultrasound generated from the protruded outer region of concave curvature first strikes the surface of quartz substrate with a full area is 30 × 10 mm^2^, see Figure 1; SPF is embedded in the quartz substrate and exposed on the same surface. Then, lateral propagation along the top surface can cause the early-arriving crosstalk in the SPFS. For the PVDF hydrophone, it was not explicitly observed in Figure 6b because the size of circular probe (1-mm diameter) is only 1/5 of the source dimension. 

**Figure 7:**
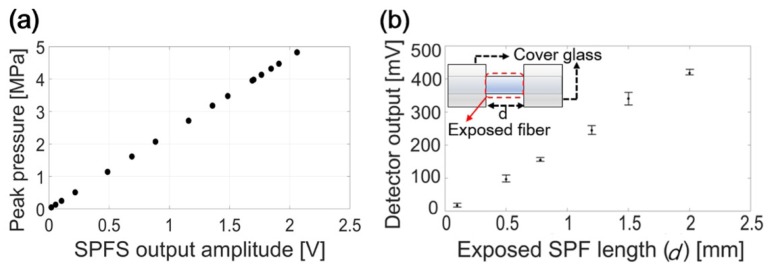
(**a**) SPFS output pressure amplitudes converted to the MPa unit (calibrated at 7-MHz frequency). An acoustic input is given from the 7-MHz piezoelectric transducer used as a source. (**b**) SPFS output signal amplitudes for different detector lengths. The inset shows a schematic to vary the exposed fiber length. An acoustic input is given from the CNT-PDMS transmitter.

The SPFS is a robust ultrasound sensor because there is no additional coating on the polished glass surface. Device robustness was tested by using high input pressure amplitudes with an order of MPa. The 7-MHz piezoelectric transducer was used as a calibrated source. In Figure 7a, the input pressure was increased up to 4.8 MPa to avoid the possible cavitation-induced damage on the SPFS. Figure 7a shows the reliable linear relationship between the SPFS output and the converted pressure amplitudes shown in the MPa unit.

The SPFS response was further investigated by varying the detector length of SPFS. The SPFS output should increase with the sensing length and thus volume. The exposed SPF length was truncated on both ends by covering them with two glass slides, only leaving a central part of the SPFS exposed and in contact with water. Because of acoustic impedance mismatch and attenuation, the glass plates effectively excluded acoustic transmission (measured reduction > 20 dB). The exposed length (d) at the center was set to 0.1, 0.5, 0.8, 1.2, 1.5 and 2 mm, corresponding to the area of 0.0008, 0.004, 0.0064, 0.0096, 0.012 and 0.016 mm^2^, respectively. For each length, the measurement was repeated multiple times by laterally moving the SPF (0.1-mm step lateral shift for d = 0.1 mm, 0.15-mm step for d = 0.5 mm, and 0.1-mm step for all other d values). Figure 7b shows measured peak pressure amplitudes for a fixed input generated by the CNT-PDMS (E = 6 mJ/pulse). This demonstrates that the signal amplitude scales linearly with the detector length. Irregular roughness formed along the sensing region can cause slight variation in each detector output. Interestingly, the SPFS with only 100-μm length still has a significant response. This suggests the design possibility of microscale detector that can be useful for miniaturized all-optical ultrasound transducers being integrated with optical transmitters such as CNT-PDMS films.

## 4. Concluding Remarks

We have demonstrated a fiber-optic ultrasound sensor using the SPF structure, featuring a broad frequency bandwidth over dc–46 MHz and a wide amplitude dynamic range of several kPa–4.8 MPa, confirmed by using photoacoustic transmitters and piezoelectric transducers as acoustic sources. The SPF uses a microscale-roughened structure for evanescent-field interaction with water that is acoustically modulated. Although the SPFS has a small detection area of only 0.016 mm^2^ with no additional coatings, it exhibits the high sensitivity of 417 and 426 mV/MPa at 3- and 7-MHz frequencies together with the NEP of 6.6 kPa (at 7 MHz) over 1–50 MHz bandwidth. Furthermore, as the sensor is operated with the bare polished surface, it allows for mechanical robustness that is suitable for reliable measurement of high-pressure amplitudes of the order of MPa. We expect that our structure and operation concept can not only be extended to the array platforms using multiple SPFs but can also be utilized to develop high-frequency and high-resolution ultrasound imaging systems, fiber integrated ultrasound endoscopes, and all-optical ultrasound transducers.
